# Formation of 3‐hydroxyglutaric acid in glutaric aciduria type I: in vitro participation of medium chain acyl‐CoA dehydrogenase

**DOI:** 10.1002/jmd2.12026

**Published:** 2019-03-26

**Authors:** Verena Peters, Marina Morath, Matthias Mack, Michael Liesert, Wolfgang Buckel, Georg F. Hoffmann, Jerry Vockley, Sandro Ghisla, Johannes Zschocke

**Affiliations:** ^1^ Center für Paediatric and Adolescent Medicine University of Heidelberg Germany; ^2^ Institut für Technische Mikrobiologie Hochschule Mannheim Germany; ^3^ Laboratorium für Mikrobiologie, Fachbereich Biologie Philipps‐Universität Marburg Germany; ^4^ Department of Pediatric and Human Genetics Children's Hospital of Pittsburgh and University of Pittsburgh Pittsburgh Pennsylvania; ^5^ Department of Biology, Section of Natural Sciences Universität Konstanz Germany; ^6^ Division of Human Genetics Medical University Innsbruck Austria

**Keywords:** 3‐hydroxyglutaric acid, acyl‐CoA dehydrogenase, glutaconyl‐CoA, glutaric aciduria type I, glutaryl‐CoA

## Abstract

3‐Hydroxyglutaric acid (3‐OH‐GA) in urine has been identified as the most reliable diagnostic marker for glutaric aciduria type I (GA I). We showed that hydratation of glutaconyl‐CoA to 3‐hydroxyglutaryl‐CoA, which is subsequently hydrolyzed to 3‐OH‐GA, is efficiently catalyzed by 3‐methylglutaconyl‐CoA hydratase (3‐MGH). We have now investigated whether mitochondrial acyl‐CoA‐dehydrogenases can convert glutaryl‐CoA to glutaconyl‐CoA. Short‐chain acyl‐CoA dehydrogenase (SCAD), medium‐chain acyl‐CoA dehydrogenase (MCAD), and long‐chain acyl‐CoA dehydrogenase (LCAD) accepted glutaryl‐CoA as a substrate. The highest *k*_cat_ of glutaryl‐CoA was found for MCAD (0.12 ± 0.01 second^−1^) and was about 26‐fold and 52‐fold higher than those of LCAD and SCAD, respectively. The turnover of MCAD for glutaryl‐CoA was about 1.5% of that of its natural substrate octanoyl‐CoA. Despite high *K*_m_ (above 600 μM) and low turnover rate, the oxidation of glutaryl‐CoA by MCAD in combination with 3‐MGH could explain the urinary concentration of 3‐OH‐GA in GA I patients.

## 1 INTRODUCTION

Glutaric aciduria type I (GA I) is an inborn error of metabolism that results from deficiency of glutaryl‐CoA dehydrogenase (GCDH), which catalyzes the oxidative decarboxylation of glutaryl‐CoA to crotonyl‐CoA.^1^, ^2^ Two biochemically defined subgroups of patients have been described based on urinary metabolite excretion of glutaric acid (GA). Most patients excrete large amounts (high excretors) of GA with urinary concentrations between 850 and 1700 mmol/mol creatinine.^2^, ^3^ However, in some GA I patients (low excretors), the urinary concentration of this compound is within the normal range of up to 4 mmol/mol creatinine.^4^ Indeed, there are some GA I patients who were identified solely by increased urinary concentrations of 3‐hydroxyglutaric acid (3‐OH‐GA), which is found in both, “high excretor” and “low excretor” patients.^5^, ^6^, ^7^ Increased levels of 3‐OH‐GA have also been found in patients with short‐chain 3‐hydroxyacyl CoA dehydrogenase (SCAD) deficiency,^8^ in patients with disorders of long‐chain fatty acid oxidation and mitochondrial disorders,^9^ and in ketotic patients.^10^ It has been suggested that 3‐OH‐glutaryl‐CoA may be produced in constant but limited amounts from accumulating glutaryl‐CoA via glutaconyl‐CoA in a two‐step process mediated by mitochondrial β‐oxidation enzymes. We previously showed that hydratation of glutaconyl‐CoA to 3‐OH‐ glutaryl‐CoA is efficiently catalyzed by 3‐methylglutaconyl‐CoA hydratase (3‐MGH).^11^ We now investigated whether mitochondrial acyl‐CoA dehydrogenases (ACADs) can convert glutaryl‐CoA to glutaconyl‐CoA, and thus, in combined action with 3‐MGH, could explain the formation of 3‐OH‐GA in GA I patients.

## 2 MATERIALS AND METHODS

Recombinant human medium‐chain acyl‐CoA dehydrogenase (MCAD) was expressed and purified as described before,^12^, ^13^ recombinant human long‐chain acyl CoA dehydrogenase (LCAD) as described by Eder et al. 1997 and short‐chain acyl CoA dehydrogenase (SCAD) as described in reference 14 butyryl‐CoA, propionyl‐CoA, hexanoyl‐CoA, octanoyl‐CoA, phenylpropionyl‐CoA, and palmitoyl‐CoA, glutaryl‐CoA were obtained from Sigma‐Aldrich (St. Louis, Missouri).

The activity of recombinant human acyl‐CoA dehydrogenases^13^ was measured at different substrate concentrations in Tris buffer with an assay using ferricenium hexafluorophosphate as an electron acceptor.^15^ Enzyme activity was additionally confirmed by identification of the product glutaconyl‐CoA by HPLC and analyzing it by mass spectrometry.^11^ The kinetic constants (*K*_m_ and *V*_max_) were estimated by computer‐fitting of the data (n = 4) using an algorithm based on the Michaelis‐Menten equation. The turnover number (*k*_cat_) was calculated using the molecular mass of the acyl‐CoA dehydrogenases (43 kDa).^16^, ^17^ In addition to glutaryl‐CoA, the activities of acyl‐CoA dehydrogenases toward their (optimal) substrates were determined as a control. The pH dependence of the activity toward the different substrates was measured in the range of pH 7.0‐8.5 in Tris buffer adjusted to the desired pH value with KOH or HCl.

## 3 RESULTS

**Figure 1:**
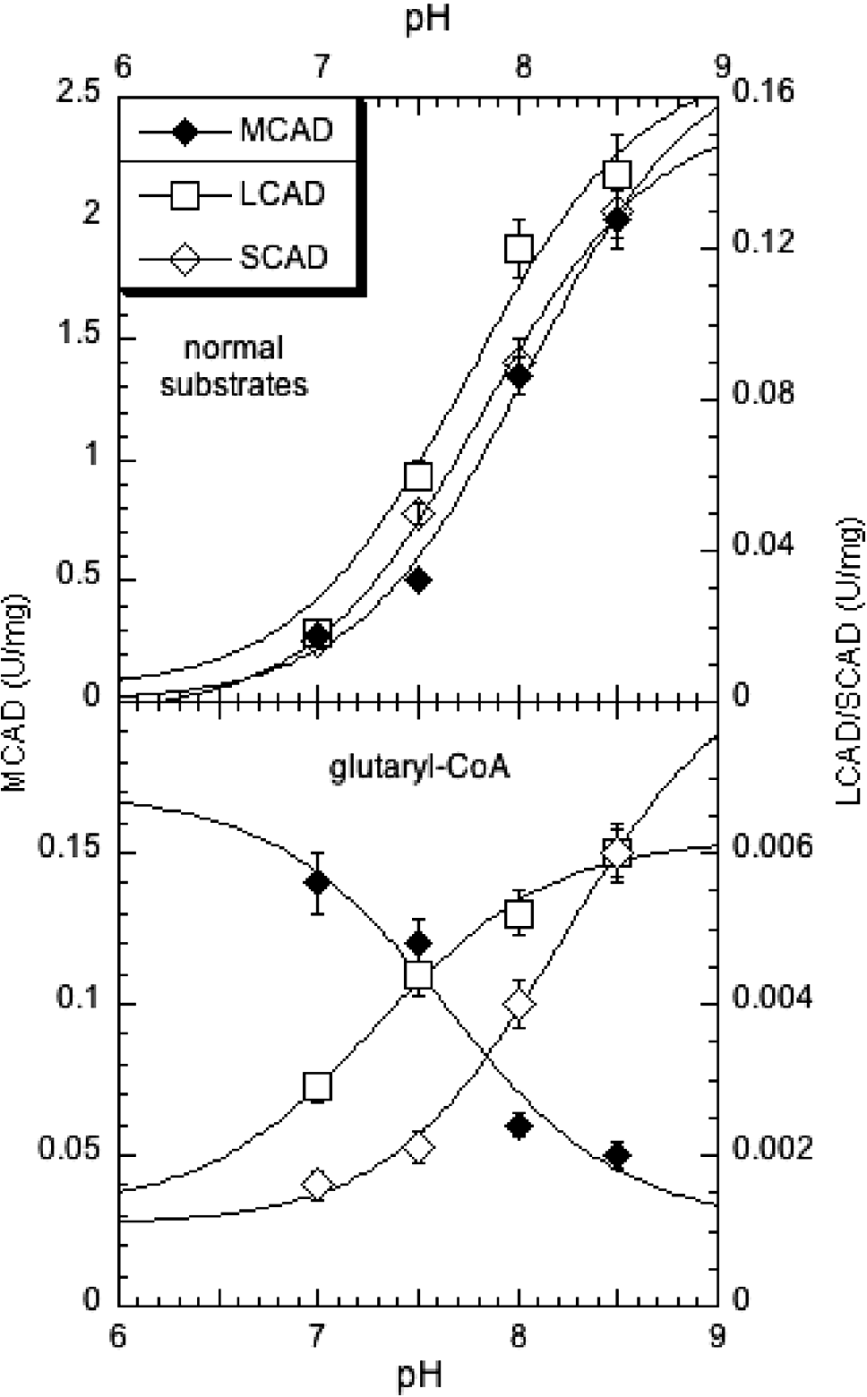
pH‐dependence of the activity of selected ACADs for glutaryl‐CoA (bottom panel) and for natural substrates as comparison (top panel). The substrates were hexanoyl‐CoA, octanoyl‐CoA, and palmitoyl‐CoA for SCAD, MCAD, and LCAD, respectively, (top panel) and glutaryl‐CoA (bottom panel). The data points are the average of four single determinations, and the vertical bars indicate the SD. For their normal substrates, the following apparent pK's MCAD: 8.05, LCAD: 7.75, SCAD: 7.85. The apparent pK's estimated for glutaryl‐CoA as substrate are MCAD: 7.6, LCAD: 7.3, SCAD: 8.2

All three ACADs were able to catalyze the dehydrogenation of glutaryl‐CoA to glutaconyl‐CoA (Table 1). The production of glutaconyl‐CoA was demonstrated by the ferricenium assay and by identification of the product by HPLC followed by mass spectrometry. At pH 7.5, MCAD exhibited the highest *k*_cat_ of 0.12 second^−1^, although about 70 times lower than with the natural substrate octanoyl‐CoA. SCAD and LCAD were much less active for the substrate glutaryl‐CoA with *k*_cat_ values of 0.0023 and 0.0045 second^−1^, respectively. Interestingly, the *K*_m_ values for glutaryl‐CoA were about equal with each enzyme (600‐900 μM), indicating that only the CoA moiety is responsible for binding to the enzyme, whereas the hydrophilic glutaryl‐CoA moiety does not interact well with the hydrophobic binding pockets. With increasing pH, the specific activities of all ACADs with their “natural” substrates increased for all three enzymes (Figure 1, top panel). In contrast, the activity of MCAD with glutaryl‐CoA at high concentration (1.2 mM) exhibited an “opposite” behavior, that is, it decreased with pH. SCAD and LCAD, on the other hand, behaved much as with their normal substrates, although the increase in activity with increasing pH was comparatively small (Figure 1, bottom panel). At pH >8.5, the difference in activities between MCAD and SCAD or LCAD per enzyme quantity was only about 5‐fold. Noteworthy is also that at lower glutaryl‐CoA concentration (<600 μM) the activities were substantially reduced for all three enzymes at all pH values.

**Table 1:** Kinetic parameters for MCAD, SCAD, and LCAD (kinetic paramters for the substrate glutaryl‐CoA are given in bold)

Enzyme	Substrate	*K*_m_ (μM)	*k*_cat_ (s^−1^)	*k*_cat_/*K*_m_ (pM^−1^ second^−1^)
**SCAD**	**Glutaryl‐CoA**	**850 ± 98**	**0.0023 ± 0.0001**	**2.7**
	Hexanoyl‐CoA	430 ± 29	0.014 ± 0.006	33
**MCAD**	Octanoyl‐CoA	6.0 ± 0.3	8.2 ± 0.4	1 360 000
	**Glutaryl‐CoA**	**660 ± 42**	**0.12 ± 0.01**	**180**
**LCAD**	Palmityl‐CoA	63 ± 4	0.143 ± 0.023	2300
	**Glutaryl‐CoA**	**650 ± 45**	**0.0045 ± 0.0001**	**6.9**

## 4 DISCUSSION

Kinetic data obtained with recombinant human MCAD, SCAD, and LCAD in the present study provide a rationale for previously unexplained biochemical findings in patients with classical and atypical forms of GA I. GA, the product of hydrolysis of glutaryl‐CoA, is usually the dominating pathological metabolite in GA I but present in highly variable concentrations. In contrast, the formation of 3‐hydroxyglutarate in GA I appears to be quite stable at approximately 0.1 mol per day and is independent from the urinary concentrations of GA (unpublished observation). Our present data indicate that the oxidation of glutaryl‐CoA in GA I is most probable catalyzed by MCAD rather than by SCAD or LCAD, with high K_*m*_ and low turnover rates, although we cannot exclude that other acyl‐CoA dehydrogenases, like isovaleryl‐CoA dehydrogenase, may also be involved in the oxidation of glutaryl‐CoA and thus may contribute to the formation of 3‐OH‐GA. Future in vivo studies and/or tracer flux analyses taking into account the complete cell metabolism should be able to provide more details on metabolic rerouting in GCDH deficiency.

**Figure 2:**
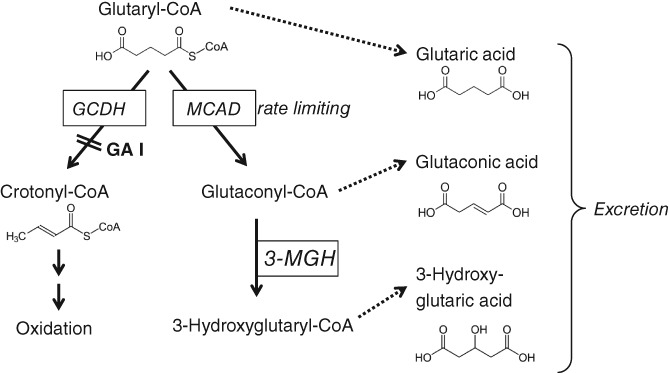
Proposed metabolic pathway of 3‐OH‐GA formation. Glutaryl‐CoA is formed within the catabolic pathway of lysine, tryptohan, and hydroxylysine. Deficient activity of glutaryl‐CoA dehydrogenase (GCDH) results in elevated levels of glutaryl‐CoA. Glutaryl‐CoA can be degraded by MCAD (and lower activity of SCAD or LCAD), and the subsequent conversion of glutaconyl‐CoA to 3‐hydroxyglutaryl‐CoA (3‐OH‐GA‐CoA) is catalyzed by 3‐methylglutaconyl‐CoA hydratase (3‐MGH^11^)

Acyl‐CoA dehydrogenases belong to the family of homotetrameric flavoenzymes with subunit molecular masses of 40 to 50 kDa^17^ that mediate the α,β‐dehydrogenation of acyl‐CoA thioesters to enoyl‐CoA in the mitochondrial β‐oxidation of fatty acids and are also involved in the degradation of leucine, isoleucine, valine, and lysine.^18^ As previously reported, the activities of ACADs are strongly pH dependent^18^ and dependent on the type of buffer used.^19^ Assuming a pH around 7.5 within the cell, the dehydrogenation of glutaryl‐CoA by MCAD (and to a much lesser extent by SCAD and LCAD) is expected to occur at a low but constant rate at concentrations in the mM range. Such concentrations are thought to be reached in the mitochondria of all (classical and atypical) patients with GA I, including those with “mild” GA1 and residual GCDH activity. Assuming that there is no “better” enzyme for this reaction, the first step in the short pathway to 3‐OH‐glutaryl‐CoA biosynthesis would be rate limiting, and turnover is not significantly affected by changes in mitochondrial glutaryl‐CoA concentrations from “very high” to “extremely high” in different variants of GA I. In “low excretor” patients with at least one hypomorphic *GCDH* mutation and residual enzyme function, the intramitochondrial elevation of glutaryl‐CoA is sufficient to lead to noticeable conversion into glutaconyl‐CoA without noticeable production of free GA. 3‐MGH efficiently converts glutaconyl‐CoA to 3‐OH‐glutaryl‐CoA with a *k*_cat_ of 1.4 seconds^−1^ 11 in a reversible reaction. After hydrolysis, the products glutaconic and 3‐hydroxyglutaric acid is excreted as free acid into the blood and finally into the urine (Figure 2).

The formation of 3‐OH‐GA in patients with SCAD or LCAD deficiency appears to be unrelated to the accumulation of glutaryl‐CoA as a precursor of 3‐OH‐glutaryl‐CoA. Molven et al.^8^ suggested that 3‐OH‐glutaryl‐CoA may be hydrolyzed to 3‐OH‐GA when the conversion of 3‐OH‐glutaryl‐CoA to 3‐ketoglutaryl‐CoA is blocked. The increased excretion of 3‐OH‐GA in ketotic patients was thought to be caused by increased protein catabolism and thus increased flux through the lysine degradative pathway.^10^

There is no urinary excretion of 3‐OH‐GA in glutaric aciduria type II (GA II), the deficiency of the electron transferring flavoprotein (ETF), which mediates the electron transport from all mitochondrial acyl‐CoA dehydrogenases (including SCAD, MCAD, and LCAD) to the respiratory chain. Instead, the excretion of 2‐OH‐GA is a useful marker for this disease.^20^ In GA II, without functional ETF as electron acceptor, accumulating glutaryl‐CoA cannot be converted into glutaconyl‐CoA, precluding the subsequent generation of 3‐OH‐GA. This observation also supports the notion that 3‐OH‐GA in GA I is generated within the mitochondria and not in another cellular compartment such as the peroxisomes.

In conclusion, we report that 3‐OH‐GA in GA I is most likely generated through MCAD‐mediated dehydrogenation of accumulating glutaryl‐CoA followed by 3‐MGH‐mediated hydratation and spontaneous hydrolysis. Throughput in this pathway appears to be not proportional to the amount of accumulating glutaryl‐CoA, explaining a constant production of 3‐OH‐GA relatively independent of residual GCDH activity. GA production, in contrast, is much more dependent on residual GCDH activity, with high excretion only in severe enzyme deficiency. Thus, 3‐OH‐GA is the most sensitive metabolic indicator of impaired GCDH function.

## AUTHOR CONTRIBUTIONS

All authors contributed to this article. Verena Peters and Johannes Zschocke were involved in planning data evaluation and writing this article. Matthias Mack, Michael Liesert, Wolfgang Buckel, Jerry Vockley, and Sandro Ghisla were involved in the experiments. Marina Morath and Georg Hoffmann critically revised the manuscript. All authors reviewed the final manuscript and gave approval for submission.

## COMPETING INTEREST STATEMENT

All authors declare that they have no conflict of interest.
